# Brimonidine-associated uveitis – a descriptive case series

**DOI:** 10.1186/s12886-020-01762-w

**Published:** 2020-12-17

**Authors:** Susanne Hopf, Karl Mercieca, Norbert Pfeiffer, Verena Prokosch-Willing

**Affiliations:** 1 Department of Ophthalmology, University Medical Center Mainz, Langenbeckstr. 1, 55131 Mainz, Germany; 2 Manchester University Hospitals NHS Trust, Manchester Royal Eye Hospital, M13 9WH, Manchester, UK; 3 Faculty of Biology, Medicine and Health School of Health Sciences, University of Manchester, Manchester, UK

**Keywords:** Brimonidine-associated uveitis, Glaucoma, Brimonidine, Anterior uveitis, Drug-induced uveitis

## Abstract

**Background:**

Anterior uveitis secondary to topical brimonidine administration is rare and not well-defined. In glaucoma patients using brimonidine, one must consider this phenomenon to avoid mis-diagnosis and over-treatment with topical steroids which in turn may increase intraocular pressure (IOP). This is the largest case series including the longest patient follow-up in the current literature.

**Methods:**

Sixteen patients (26 eyes) with consultant diagnosed brimonidine-associated anterior uveitis in a tertiary referral glaucoma clinic presenting between 2015 and 2019 were included in this retrospective case series. Clinical records were taken for descriptive analysis. Main outcome measures were the key clinical features, and disease course (therapy, IOP control, patient outcome).

**Results:**

Key features were conjunctival ciliary injection and mutton fat keratic precipitation in all eyes. The findings were bilateral in 10 patients. Time between initiation of brimonidine treatment and presentation was 1 week to 49 months. Glaucoma sub-types were mostly pseudo-exfoliative and primary open angle glaucoma. Brimonidine treatment was stopped immediately. Additionally, topical corticosteroids were prescribed in 18 eyes and tapered down during the following 4 weeks. Thirteen eyes did not need surgical or laser treatment (median follow-up time 15 months). No patient showed recurrence of inflammation after cessation of brimonidine.

**Conclusions:**

This type of anterior uveitis is an uncommon but important manifestation which should always be considered in glaucoma patients on brimonidine treatment. Although treatable at its root cause, problems may persist, especially with respect to IOP control. The latter may necessitate glaucoma surgery after the resolved episode of the uveitis.

**Supplementary Information:**

The online version contains supplementary material available at 10.1186/s12886-020-01762-w.

## Background

Brimonidine tartrate, a selective α2-agonist, is often administered as second line therapy or as part of a combined topical regime in glaucoma and ocular hypertension [1]. The granulomatous anterior uveitis associated with its use was first described by Byles in 2000 [2]. A cumulative total of 39 cases (68 eyes) within 15 reports [1–16] have been published since then, the greatest number previously reported being 12 cases (19 eyes) in 2015 [4]. To the best of our knowledge, this is the largest case series of brimonidine associated uveitis, including the longest patient follow-up in the literature.

Brimonidine-induced uveitis with corneal endothelial inflammatory deposits (keratic precipitations or KPs) and usually cells and flare, occurs in elderly patients, mostly above the age of 75 years, and following several years of brimonidine application [10]. Most authors recommend stopping brimonidine use with or without the administration of steroid [12] and/or non-steroidal anti-inflammatory eye drops.

Very little is known about the specific characteristics of patients developing this uncommon type of drug-induced anterior uveitis. However, one must consider this phenomenon to avoid mis-diagnosis and over-treatment with topical steroids which in turn may increase intraocular pressure. The aim of our study was to try and identify the key clinical features of this condition and to look for possible co-factors such as duration of brimonidine treatment and sub-type of glaucoma. In view of the paucity of evidence on how to treat this type of uveitis, our second aim was to report on the disease course with respect to therapy whilst also focusing on intraocular pressure (IOP) control and clinical outcomes.

## Methods

This is a retrospective descriptive case series of 16 consecutive patients (26 eyes) diagnosed with brimonidine-associated anterior uveitis in a tertiary referral glaucoma clinic presenting between 2015 and 2019. The diagnosis was made by a consultant glaucoma sub-specialist, and any unconfirmed or doubtful cases with regards to uveitis causation were excluded.

Clinical records were accessed for descriptive analysis. We annotated anterior segment examination (including the evaluation of inflammation according to the SUN grading system), initiation of brimonidine, current topical medication, IOP and visual acuity, type of glaucoma and further ocular diseases, previous surgeries, history of systemic inflammatory disease (e.g. rheumatoid arthritis), subsequent therapy regimens, and clinical outcome (IOP, eventually surgery, inflammation). Patient characteristics were categorized as absolute and relative frequencies for categorical variables, and as mean and standard deviations for continuous variables.

Criteria for the diagnosis were: anterior uveitis in one or both eyes for no other reason than the use of brimonidine or brinzolamide-brimonidine fixed combination topical therapy. Keratic precipitation and eventually cells or flare in association with these drops, in the absence of systemic inflammatory or infectious disease, were considered as suspicious, even more so in combination with elevated IOP. The selected cases did not undergo a conjunctival biopsy prior to diagnosis as this is not pathognomonic [5]. Serologic and radiological workup was performed in cases where it was deemed indicated (for example bilateral involvements), and included routine laboratory testing, c-reactive protein, differential blood count, antinuclear antibodies, angiotension converting enzyme, soluble IL-2 receptor, HLA B 27, infectious laboratory (tuberculosis, syphilis, borrelia), and chest x-ray.

## Results

We identified 26 eyes in 16 consecutive patients with brimonidine-associated uveitis, with a male-to-female ratio of 7:9 and a mean patient age of 76 years (standard deviation (SD) +/− 10 years). The brimonidine ‘exposure time’ spanned from 1 week to 49 months (median 15 months) and was defined as the time between the documented start of brimonidine treatment and the clinical diagnosis of the drug-induced anterior uveitis. The exposure time was based on available data from 10 patients (14 eyes), and was unknown for 6 patients (12 eyes). Detailed patient data is provided in the Table 1.

**Table 1:** Characteristics of the case series: Predisposing factors and presenting symptoms

*ID*	*Age, Gender*	*Eyes affected*	*Uveitis signs (conjunctival hyperemia present in all affected eyes)*	*Glaucoma therapy*	*Brimonidine Use*	*Type of glaucoma*	*Prior surgery*	*Auto- immune or rheumatoid disease*	*Iris pigmentation*	*logMAR visual acuity R/ L*
1	80’s-90’s, f	Both	KP, cells 1+ (R + L), Synechia (R)	Both: Br	13 mo.	NTG	No	No **	Dark	0.6 0.4
2	60’s-70’s, m	Both	KP, cells 1+ (R + L), synechia (R)	Both: Br, DoTim, Tra	> 4 mo.	OHT	TE, N, 4x CPC, Psph (14 mo.) (R); 4x CPC (L)	Crohn’s disease *	Dark	0.0 0.0
3	80’s-90’s, f	Both	unpigm. KP and cells 1+ (R + L), synechia in the chamber angle (L)	Both: Br, BzTim	n.a.	NTG	Psph (8 mo.) (R); Psph (7 mo.) (L)	No *	–	0.5 0.1
4	70’s-80’s, f	Both	KP, flare 2+ (R + L)	Both: Br, Do, BimTim, Pil, Acet	n.a.	POAG	Psph (3 years) (R); Psph (3 years) (L)	No	Dark	0.6 0.5
5	60’s-70’s, m	Left	KP (L), flare 2+ (R + L)	Both: Br, BimTim	> 24 mo.	POAG	LTP(R); LTP(L)	Hay fever	Pale	0.0 0.1
6	70’s-80’s, f	Both	KP (L)	Both: Br, DoTim, Taf, Pil, pred	n.a.	PXG	Psph (n.a.), XEN, 2x N (R); Psph (n.a.), 2x XEN, PPV, cyclocryocoagulation (L)	Intermediate uveitis 2 years earlier	Pale	0.22 0.7
7	80’s-90’s, f	Left	KP (L)	Both: Lat; Left: BzBrim	2 mo.	POAG	Psph (n.a.), cyclocryocoagulation (R); Psph (n.a.) (L)	No	Pale	0.5 0.6
8	80’s-90’s, m	Both	KP (R > L)	Both Br, DoTim	6 mo.	PXG (R); PXG - > sec. Glaucoma ^^ (L)	Psph (4 mo.) (R)	No	Pale	0.22 3
9	80’s-90’s, m	Left	KP (L)	Both: Do, Taf; Left: Br	31 mo.	PXG	Psph (3 years), TE, multiple AntiVEGF-IVOMs (R); TE, TE-Revision, Psph (3 years) (L)	No	Pale	0.22 0.22
10	70’s-80‘s, m	Right	KP, DF, cells 2+, flare 2+ (R)	Both: Br, DoTim	18 mo.	POAG - > sec. Glaucoma ^ (R); POAG (L)	Psph (1 year) (R); Psph (1 year) (L)	No **	Pale	0.6 0.4
11	40’s-50’s, m	Left	–	Both: Br, BzTim	> 49 mo.	sec. Glaucoma	AC-IOL (R); AC-IOL, 2x PPV, AC-IOL-Expl. + Psph (4 years), 2x steroidal IVOMs, CPC (L)	No *	–	0.4 1.0
12	70’s-80’s, f	Right	whitish KP (R), vascularized cornea (L)	Both: BzBrim, BimTim	24 mo.	POAG	Psph (n.a.) (R); Aphak (n.a.) (L)	No	Pale	0.3 3
13	70’s-80’s, f	Both	large whitish KP (R + L)	Both: BzBrim, Lat	n.a.	PXG	No (R); Psph+iStent+TA (9 mo.) (L)	n.a.	–	0.4 1.8
14	70’s-80’s, f	Both	KP (R < L), cells 1+, flare 2+	Both: Br, LatTim	0.25 mo.	PXG	Psph (n.a.) (R); Psph (n.a.), TE (L)	Hay fever, allergic asthma **	Dark	0.6 3
15	80’s-90’s, f	Both	KP (R + L), cells 2+, flare 2+	Both: Br, DoTim, Lat, BzTim	n.a.	PXG	Psph (1 year) (R); Psph (1 mo.), TE, N (L)	No	Dark	0.3 0.22
16	80’s-90’s, m	Both	KP (R + L)	Both: Br, BzTim	n.a.	PXG	Psph (1.5 years) (R); Psph (4 years) (L)	No	–	2.3 2.3

*Acet* acetazolamide, *AC-IOL* anteror chamber intraocular lens implantation, *BimTim* bimatoprost/ timolol fixed combination, *Br* brimonidine, *BzBrim* brinzolamide/ brimonidine fixed combination, *BzTim* brinzolamid/ timolol fixed combination, *CPC* Cyclophotocoagulation, *Do* dorzolamide, *DoTim* dorzolamide/ timolol fixed combination, *Expl*. Explantation, *iStent* iStent® implantation, *IVOM* intravitreal operative medication, *Lat* latanoprost, *LatTim* latanoprost/timolol fixed combination, *LTP* lasertrabeculoplasty, *mo*. month(s), *N* Needling, *n.a*. not applicable, *NTG* normal tension glaucoma, *OHT* ocular hypertension, *Pil* pilocarpine, *POAG* primary open angle glaucoma, *PPV* pars plana vitrectomy, *pred* prednisolone acetate, *Psph* cataract surgery, *PXG* pseudoexfoliative glaucoma, *TA* trabecular meshwork aspiration, *Taf* tafluprost, *TE* trabeculectomy, *Tra* travoprost, *XEN* XEN® stent implantation. (*) medical evaluation (completely **) done (routine, infectious, and autoimmune laboratory). (^) initial differential diagnosis was phacomorphic keratopathy, in follow-ups after 2 months hyperemic iris vessels were classified as determined by uveitis, after 4 months rubeosis was identified correctly to be due to (^^) the development of ocular ischemia

Glaucoma sub-types were pseudo-exfoliative glaucoma (7), primary open angle glaucoma (5), normal tension glaucoma (2), and secondary glaucoma (1) while one patient had ocular hypertension. Two patients subsequently developed ocular ischemia, one case in the affected eye, the other on the contralateral side. The key features documented for all 26 eyes were ciliary injection and pigmented (brownish) (Supplemental Fig. 1) or unpigmented (Supplemental Fig. 2), granulomatous type, “mutton fat” KPs. Intraocular inflammation with cells were detected in 11 eyes (42%), flare in 8 eyes (31%) and posterior synechiae in 3 eyes (12%). Posterior segment involvement was not present.

Serological diagnostic workups were performed in six patients (three of them received a complete workup as mentioned above) (see Table 1, marked by an asterisk) but did not give indications of any underlying pre-disposing inflammatory conditions. No relevant systemic illness such as inflammatory conditions etc. were present in our cohort, although four patients (25%) did have previous history of had hay fever, asthma, Crohn’s disease or intermediate uveitis. All bilateral cases (10) were on brimonidine treatment to both eyes. In one patient, the fellow eye could not be assessed for uveitis due to corneal scarring and vascularization and this patient was classified as a unilateral case. Two of the six unilateral cases were treated with brimonidine solely to the affected eye while the remaining four were having bilateral brimonidine application but did not develop uveitis in the fellow eye during the study period. Cataract surgery had been performed 1 month to 4 years prior to onset of uveitis in 20 out of 26 eyes (77%) (Table 1). None of those patients had evidence of iris transillumination. A dark iris was present in five patients while a pale (blue/green) iris was described in seven patients. The remaining four patients had no specific description of their iris pigmentation. At the time of diagnosis, median logMAR visual acuity in affected eyes was 0.45 (min. 0.0, max. 3.0), and mean IOP was 26.6 (SD +/− 11.3) mmHg. 62% (16 out of 26) of the affected eyes showed IOP above 20 mmHg. Thirteen patients (81%) were on brimonidine 0.2% (Alphagan®, Allergan) as a stand-alone drug with the other three using brimonidine within a fixed combination of brinzolamide 1.0% and brimonidine 0.2% (Simbrinza®, Alcon). Prostaglandins were being used by 10 patients in 17 eyes (65%).

All patients were told to stop brimonidine treatment in the affected eye(s), and 11 patients (18 eyes, 69%) were additionally advised treatment with a short course of topical steroids (4 times daily initially) tapering it down over the following 4 weeks (i.e. 69% of the affected eyes received steroids); see Table 2. Re-challenges to confirm the cause of uveitis were not performed as per the patients’ wishes. At a minimum follow-up time of at least 1 month (median 15 months), uveitis resolution was rapid and complete in all affected eyes, with no recurrence after the investigated follow-up time.

**Table 2:** Characteristics of the case series: IOP course and management

*ID*	*Age*	*Type of glaucoma*	*Eyes affected*	*Management: (All stopped Brimonidine)*	*IOP R/L (mmHg)*	*IOP R/ L (mmHg) course and surgical management if needed at follow-up*	*Follow-up time*
1	80’s-90’s	NTG	Both	steroids (pred)	20 15	14 15	84 mo.
2	60’s-70’s	OHT	Both	steroids (dexa, flu)	21 22	18 14	14 mo.
3	80’s-90’s	NTG	Both	steroids (dexa)	42 35	6 with Acet 6 with Acet	1 mo.
4	70’s-80’s	POAG	Both	steroids (dexa)	35 41	34 - > TE –> 13 after 2x N 34 - > TE –> 7	28 mo.
5	60’s-70’s	POAG	Left	steroids (dexa)	(13) 15	(12–16) 12–16	2 mo.
6	70’s-80’s	PXG	Both	–	41 24	12 after TE - > 10 34 –> 34	1 mo.
7	80’s-90’s	POAG	Left	steroids (dexa)	(13) 17	(15 - > CPC - > 10) 24 - > CPC 10	1 mo.
8	80’s-90’s	PXG (R); PXG - > rubeotic secondary glaucoma ^^ (L)	Both	–	31 59	10–18 26–45 with Acet	3 mo.
9	80’s-90’s	PXG	Left	steroids (dexa)	(15) 22	(22 - > 12) 31 –> CPC - > 16	9 mo.
10	70’s-80’s	POAG - > rubeotic secondary glaucoma ^ (R); POAG (L)	Right	steroids (pred)	24 (10)	13 - > 45 - > 2x CPC - > 36 (10 - > 20 - > 19)	7 mo.
11	40’s-50’s	secondary glaucoma	Left	–	(18) 29	(18 - > 10) CPC - > 23 - > 21	12 mo.
12	70’s-80’s	POAG	Right	–	28 (20)	19–22 (6–16)	1 mo.
13	70’s-80’s	PXG	Both	–	13 27	CPC 16 16 (with Acet)	0.5 mo. (lost to follow-up)
14	70’s-80’s	PXG	Both	steroids (dexa)	15 15		(lost to follow-up)
15	80’s-90’s	PXG	Both	steroids (flu)	30 17	30 - > TE - > 8 17 - > 17	1 mo.
16	80’s-90’s	PXG	Both	steroids (dexa)	18 15	5 9	1 mo.

*Acet* acetazolamide, *CPC* Cyclophotocoagulation, *dexa* dexamethasone, *flu* fluorometholone, *mo.* month(s), *N* Needling, *n.a*. not applicable, *NTG* normal tension glaucoma, *OHT* ocular hypertension, *POAG* primary open angle glaucoma, *pred* prednisolone acetate, *PXG* pseudoexfoliative glaucoma, *TE* trabeculectomy. (^) initial differential diagnosis was phakomorphic keratopathy, in follow-ups after 2 months iris hyperemia was classified as determined by uveitis, after 4 months rubeosis was identified correctly to be due to (^^) the development of ocular ischemia

In 13 eyes, IOP was well regulated without surgical intervention (see Table 2); another 2 eyes were lost to follow up. Surgery for IOP control was eventually required in 9 eyes (4 eyes underwent subsequent trabeculectomy and 5 eyes a cyclodestructive procedure), while 2 eyes were untreated for elevated IOP due to the poor prognosis (visual acuity was No Light Perception in one case and in the other case the fellow eye had already previously undergone trabeculectomy as this was the better seeing eye).

## Discussion

To the best of our knowledge, this study presents the largest case series and longest follow-up of brimonidine-associated anterior uveitis in the literature. With regard to our investigated cohort, four key findings may be highlighted, but may change within a larger population: Firstly, the time between initiation of brimonidine treatment and uveitis presentation varied widely between 1 week and 49 months. Secondly, the clinical features were mainly ciliary injection and mutton fat keratic precipitates while cells, flare and posterior synechiae were less frequent; IOP was elevated in the majority of patients (two thirds of eyes). Thirdly, glaucoma sub-types were primarily pseudoexfoliative and primary open angle glaucoma. Finally, within a month of follow-up, IOP was well-controlled in half of the affected eyes (which included 6 of the 16 eyes which had prior IOP elevation above 20 mmHg), without the need for surgery or laser treatment. A procedure was eventually needed in nine eyes, four undergoing trabeculectomy and 5 a cyclodestructive procedure.

The time interval between initiation of brimonidine treatment and presentation varied widely. The patients in our case series had been using brimonidine from just over one week up to four years prior to onset of symptoms and diagnosis. This is in line with the indicated 1 week to 5 years interval in the report from Beltz et al. [4]. Based on the lengthy spell of time between first administration and appearance of the uveitis in many cases, it is presumed that besides a primary toxic reaction, a secondary cell-mediated immune response may also be involved in the patho-physiology with the drug acting as a potential antigen or immune modulator [17]. In patients on long-term treatment, it has been postulated that inflammatory cytokines within the vitreous humor may contribute to granulomatous uveitis [18].

In our series, an elevated IOP accompanied the anterior segment reaction in the majority of cases (62% of eyes). In contrast, eight eyes (42%) in the cases from Beltz et al. showed an IOP rise of 5 mmHg or more from baseline and seven eyes (36%) were estimated too high necessitating further glaucoma therapy [4]. In our series, 9 out of 26 (35%) eyes underwent laser or surgical intervention.

Clinical signs may differ and there is no pathognomonic appearance as one finds, for example, in Fuch’s uveitis syndrome. The previously described clinical spectrum of brimonidine-associated uveitis ranges from simple conjunctival congestion [13], severe follicular conjunctivitis, blepharitis [11, 14, 15], and punctate corneal epithelial erosions [5], to the full extent of anterior uveitis with corneal endothelial deposition (mutton fat KPs) [4, 6], significant anterior chamber activity (cells and flare) [3, 4, 9] and posterior synechiae (PS) [1, 2, 7]. PS were present in only three of our patients, while Beltz et al. did not find any in their series, despite reporting cells and flare in most cases [4]. We detected scattered and mostly small mutton fat KPs in nearly all cases with large whitish KPs in only one case; Beltz et al. mostly found stellate KPs [4]. Our cases had a mean age of 76 years (SD +/− 10 years), and were thereby comparable with Beltz et al.’s series where mean age was 74 years (SD (+/− 9 years) [4].

In our cohort brimonidine-associated uveitis seemed to occur mostly in patients with pseudoexfoliative and primary open angle glaucoma. Although this may be a simple reflection of the relative frequency of these two sub-types, pseudoexfolation syndrome may itself occasionally present with keratic precipitates as well, masquerading as uveitis. The appearance was reported as white dandruff-like flakes diffusely distributed over the endothelial surface which do not disappear after topical corticosteroid administration and which are not accompanied by intraocular inflammation [19].

The coexistent elevated IOP could be a result of a hypertensive uveitic response but could also be due to an overall sub-optimal IOP control which necessitated the use of brimonidine in the first place. It remains unclear whether previous surgery (particularly cataract surgery) might play a role in the development of brimonidine-associated uveitis. Beltz et al. included 16 pseudophakic eyes (out of 19 total eyes) and postulated that patients undergoing cataract surgery were often in the same age as patients with glaucoma. According to them, one could hypothesize that the damaged corneal integrity due to phaco-incisions with drugs penetrating or diffusing into the anterior chamber resulting in intraocular reaction [4]. We find this concept unlikely, especially the longer the time lapses from the surgery, as wound healing and corneal recovery would have occurred. It is more likely that previous cataract surgery may have weakened the blood-aqueous barrier resulting in a lower anterior chamber inflammatory threshold. The calculation of statistical associations was not possible due to the small and heterogenous sample size.

As brimonidine accumulates in pigmented tissues, one could expect eyes with dark irides to be more prone to this type of drug-induced uveitis [5]. However, a larger proportion of patients in our study exhibited pale irides, thus counteracting this argument. Possible confounding factors for acute anterior uveitis in our cohort could include other local agents such as prostaglandin analogues (used in 16/26 eyes), benzalkonium chloride (present in brimonidine eye drops), topical beta-blockers and carbonic anhydrase inhibitors, and systemic drugs [20–22]. Previous operations might have also predisposed to hypersensitivities to various agents, lowering the sensitivity threshold for the inflammatory trigger caused by brimonidine. In one case, we diagnosed a case of brimonidine-associated uveitis which subsequently developed secondary neovascular glaucoma due to ocular ischemia. The latter can also present clinically with signs of anterior ocular inflammation although in our case the uveitis had occurred prior to and had completed subsided by the time neovascularization developed. Oli and Joshi presented one case of neovascular glaucoma due to central retinal vein occlusion developing brimonidine-associated ‘uveitis’ with conjunctival congestion and mild chemosis but without intraocular inflammation [13]. We therefore recommend to look carefully for iris/angle neovascularization, and if necessary retinal ischemia, in any patient with suspected brimonidine-associated uveitis. The second patient with ocular ischemia in our cohort presented with KPs predominantly on his right eye, while the ischemia affected his left eye only.

Our analysis showed complete and rapid resolution of uveitis when brimonidine itself was stopped in isolation (one third) or when this was combined with a short administration of topical steroid (two thirds). Our treatment strategy in managing uveitis with coexistent elevated IOP was correlated with the severity of inflammation and degree of glaucomatous optic nerve damage. In cases of corneal changes in isolation (i.e. KPs), we stopped brimonidine alone, especially when pseudoexfoliation syndrome was present and might contribute to the corneal abnormalities, while topical steroid might be considered when a more diffuse and/or severe inflammatory element was present.

The potential effects of steroid administration may of course include the masking of other etiologies such as autoimmune or viral uveitis. Steroids can also further increase IOP, although this commonly increases over weeks rather than days. In our cohort, steroid administration did not further increase IOP. As none of the patients reported steroid response in the history, and IOP was elevated before steroid commencement, we conclude that the reason in most cases with persistent uncontrolled IOP was not a steroid response.

Although inflammation was resolved in all eyes, nine eyes needed surgical IOP control. The elevated IOPs were not exclusively due to the uveitis but probably more due to sub-optimal IOP beforehand which could represent an uncontrolled course of the glaucomatous process, partly due to the adverse effect of brimonidine itself. In patients who required surgery, the IOP was managed early and effectively after the resolution of the uveitis.

Ophthalmologists should consider brimonidine-associated uveitis as reversible uveitis syndrome. Conscious acquaintance with this condition enables waiving needless tests and therapies [4]. In unclear cases of uveitis with KPs and eventually cells and flare in glaucoma patients on topical brimonidine, the manifestation of brimonidine-associated uveitis should be always considered. Stopping brimonidine usually resolves and prevents recurrence of the inflammation; however, the subsequent management of high IOP may result in the need for surgery.

## Supplementary Information

**Additional file 1: Supplemental Figure 1.** Anterior segment photograph showing pigmented keratic precipitates (ID 15).

**Additional file 2: Supplemental Figure 2.** Anterior segment photograph showing scattered, mostly depigmented keratic precipitates (ID 14).

## Data Availability

All data generated or analysed during the current study are included in this published article (presented in Tables 1 and 2).

## Abbreviations

KP: Keratic precipitations
IOP: Intraocular pressure
PS: Posterior synechiae
