# Supplementary figures and images for: Brimonidine-associated uveitis – a descriptive case series
Source: BMC Ophthalmol. 2020 Dec 17;20:489. doi: 10.1186/s12886-020-01762-w (PMC7745535; doi:10.1186/s12886-020-01762-w)

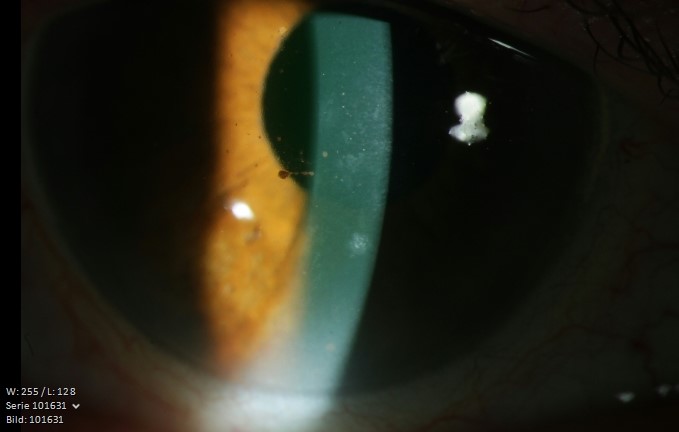

Supplement: Supplementary file 1 — Additional file 1: Supplemental Figure 1. Anterior segment photograph showing pigmented keratic precipitates (ID 15). [file 12886_2020_1762_MOESM1_ESM.jpeg]

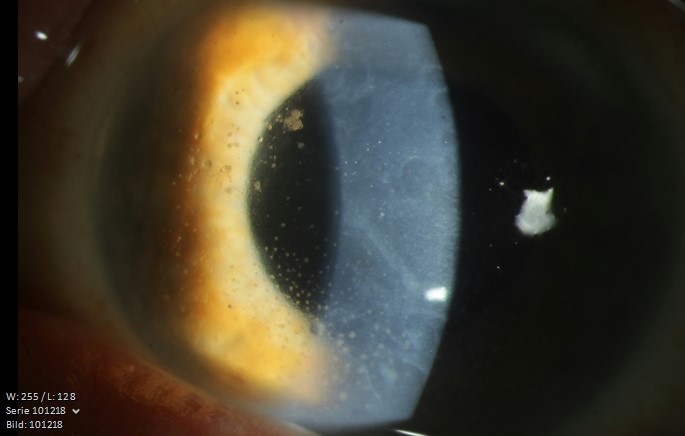

Supplement: Supplementary file 2 — Additional file 2: Supplemental Figure 2. Anterior segment photograph showing scattered, mostly depigmented keratic precipitates (ID 14). [file 12886_2020_1762_MOESM2_ESM.jpg]
